# Reconciling relationships with physical activity: a qualitative study of women’s postnatal physical activity decision-making

**DOI:** 10.1186/s12884-020-03537-z

**Published:** 2021-01-25

**Authors:** Sarah Jane Liva, Wendy Anne Hall, John Oliffe

**Affiliations:** 1 School of Nursing, Trinity Western University, 7600 Glover Road, Langley, BC V2Y 1Y1 Canada; 2 School of Nursing, University of British Columbia, T201 2211 Wesbrook Mall, Vancouver, BC V6T 2B5 Canada

**Keywords:** Physical activity, Postpartum, Decision-making, Grounded theory, Exercise

## Abstract

**Background:**

Challenges with engaging in postnatal physical activity can negatively affect the health of women and their families. This study investigated women’s physical activity decision-making processes and strategies to support their physical activity as part of a healthy postpartum transition.

**Methods:**

Thirty healthy women with infants aged 2.5–12 months completed 3-day activity diaries and an individual interview. Using Glaser and Charmaz’s grounded theory methodology, the core category, reconciling relationships with physical activity, was constructed, which explained women’s processes of postnatal physical activity decision-making.

**Results:**

Through reconciling relationships with physical activity, women discerned the types of physical activity they were comfortable pursuing at various points in the postpartum transition. Based on the meaning physical activity held for participants and their views about risks, supports, and resources, women gauged their capacity and the workability of their physical activity desires. Most women were uncertain of their capacity (physical, emotional) to return to physical activity and viewed the achievement of several or all of their desired physical activities as unworkable. Only a small group of women fully pursued the desirable physical activities they viewed as important for their well-being. Women adjusted the strategies they used to achieve physical activity when their expectations of capacity and workability did not align with their experiences. Some women lacked access to resources or supportive messaging about postpartum physical activity and downgraded their physical activity pursuit after negative personal physical or childcare experiences.

**Conclusions:**

Women can benefit from discussions about physiological birth recovery and navigating community and peer resources to support physical activity access and the safe return to physical activity following birth.

**Supplementary Information:**

The online version contains supplementary material available at 10.1186/s12884-020-03537-z.

## Background

Stress during the year following childbirth (i.e., the postnatal period) can increase women’s vulnerability to negative mental health outcomes, decreased relationship quality, disordered eating, and weight gain [1]. Physical activity enhances women’s general well-being, sleep quality, partner relationships, and physical recovery from birth [2, 3]. Women who have engaged in exercise interventions have reported fewer depressive symptoms [4]. Maternal depressive symptoms, even if they do not reach clinical levels, have been associated with children’s long-term development of emotional-behavioral difficulties [5]. Thus, maternal physical activity may also support healthy infant development. Previous findings highlight that the likelihood of achieving physical activity recommendations (30 min daily moderate physical activity) decrease for women following birth [6]. Canadian women with young children exercise 7 min less per day than women without children [7]. The majority of participants in large descriptive studies measuring women’s postpartum physical activity did not achieve physical activity recommendations [8, 9]. Qualitative descriptive and survey research has illuminated personal, relational, and resource factors (e.g., facilitators, barriers) that women perceived as affecting their postnatal physical activity choices [10–12]. Women have identified lack of time/childcare, fatigue, and social support as significant factors affecting their postnatal physical activity levels [11, 13]. Interventions targeting these factors to support postpartum physical activity have demonstrated mixed success [14]. A meta-analysis found that postnatal physical activity interventions had a short-term moderate-sized effect on women’s activity frequency but no effect on overall activity volume [14].

A qualitatively-derived theoretical model explaining women’s physical activity decision-making following birth could contribute to identifying effective strategies to support activity, in part, by explaining how varying influences affect women’s choices and physical activity engagement. Qualitative research describing parents’ diverse rationales for physical activity engagement point to significant complexity in ways women construct their physical activity choices [15]. Evidence suggests women’s postpartum physical activity trajectories vary; the largest percentage of women decrease physical activity but some women maintain previous activity and a small percentage increase their physical activity in the postpartum period [16]. Women have described incorporating social expectations and taking cues from their physical body and past exercise experiences to make physical activity decisions prenatally and in the early postpartum period [17]; however, previous work has not explicated complexity and variation in women’s physical activity choices at various points in the postpartum period. This qualitative study explains women’s postnatal physical activity decision-making processes. We used constructivist grounded theory design to understand patterns of behavior and conditions that create variability in decision-making [18].

## Methods

Our research design used Glaser’s [19] approach and incorporated Charmaz’s [18] constructivist grounded theory methodological considerations to account for the relational and constructivist nature of qualitative inquiry.

### Participants and procedure

We obtained ethical approval for this study from the University Behavioral Research Ethics Board. Thirty healthy women (between 2.5–12 months postpartum) who had habitual risk pregnancy were recruited from three mid-sized Canadian cities.

Recruitment continued until theoretical saturation, when new data did not contribute to further developing category properties or their interrelationships [18]. Flyers were posted on social/print media (e.g., Facebook groups, magazines) and in community spaces (e.g., library, physician offices). Consistent with grounded theory methodology we used purposive and theoretical sampling. We initially purposively recruited women who were most likely to provide rich data [18]. As our analysis progressed we moved to theoretical sampling. Theoretical sampling involves sampling participants in response to the emerging categories to further theory development [18]. After we had generated preliminary data and categories, we sampled theoretically to refine emerging categories and their interrelationships. For example, in early interviews we found women who were using childcare reported fewer barriers to physical activity. In response to this data, and to diversify understanding about childcare use and the personal beliefs category we posted flyers at fitness centres with childcare to specifically recruit women using those facilities.

The average participant age was 33.6 (range 26–43). Infants were between 2.5–12 months (average 6.3 months). As shown in Table 1, most participants were married and/or had university degrees. Half of the women had one child and/or a combined family income of more than $100,000; 83% were on maternity leave. More than half of the participants self-identified as Caucasian. Of the five women not on maternity leave, four were working part-time, and one woman identified her primary occupation as caring for her children at home.

**Table 1:** Characteristics of the Sample

Characteristic	Number
Relationship status	
Living with partner	29
Single	1
Number of children at home	
1	15
2	9
3	6
Education	
University degree	25
Some college	3
High school	2
Income (combined family)^ab^	
$ > 100,000	16
$80,000 – < $100,000	5
$60,000 – < $80,000	4
$20,000 – < $40,000	3
Ethnicity^ac^	
Caucasian and/or white	17
Other (European or Iranian or Korean or Chinese)	6
Mixed or biracial	2
Canadian	3
Occupation	
Professional e.g., teacher	17
Industry e.g., marketing	9
Student	3
Caring for child at home	1

^a^Two women did not answer. ^b^No women identified income between $40,000 – $60,000. ^c^Women had the option to self-identify ethnicity. Many Canadians prefer to identify as Canadian rather than specifying a country of origin or racial identity

Women gave informed consent prior to interviews and received a coffee card as thanks for participating. The first author (SL) collected interview and diary data from September 2014 to 2015. Women completed 3-day diaries prior to interviews with SL, to promote their reflections about physical activity decision-making and support interview discussions. In their diaries, women listed hourly activities and described activity contexts, e.g., environmental noise levels, and personal experiences of activities e.g., their emotions. Interviews were audio-recorded and transcribed verbatim, with identifiers removed. They ranged from 43 to 75 min in length. SL provided two participants who became distressed during their interviews with appropriate resources and follow-up.

Early interviews focused on exploring participants’ perspectives and the meanings they attached to their postnatal physical activity experiences (Supplementary file 1). Women started the conversation by sharing their thoughts about their activity diaries and SL explored ideas the women expressed (e.g., concern about sleep).

Theoretical sensitivity, the ability to draw connections between codes to develop a theory [19], was enhanced because SL was sensitized to concepts (personal, social, policy, and environmental factors) from the literature related to physical activity decision-making [20]. SL asked participants to elaborate about the meaning and significance of such concepts as they spoke about them [19]. Later interviews incorporated theoretical diagrams and more focused discussion to elaborate and develop categories and their relationships to the core category [18]. Participants commented on preliminary findings in 2015.

### Data analysis

Data collection and analysis were concurrent, and constant comparison techniques were used to compare diary and interview data [19]. Data were open-coded until the core category was identified (by interview 23); data were selectively coded to delimit them around the core category and theoretically coded to integrate the theory [19]. Reflexive journaling, theoretical diagramming, and extensive memoing continued throughout analysis. SL shared analytical ideas, diagrams, and memos throughout the analytic process with co-authors for feedback. To support reflexivity and relationality, SL used journaling and peer debriefing [18]. Glaser’s criteria for [19] rigor rely on fit, work, relevance, parsimony, and modifiability. Constant comparison and coding processes supported the development of a core category that fit the data and accounted for maximum variation in behavior; it also focused the theory to support parsimony, the fewest number of categories to provide the greatest level of theoretical explanation [19]. Being reflexive supported the development of a theory that was relevant to and reflective of participants’ perspectives.

## Results

**Fig. 1:**
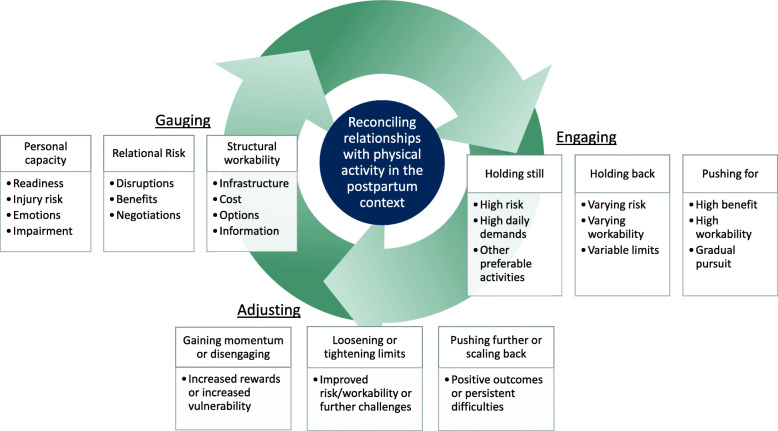
Diagram illustrating phases of reconciling relationships with physical activity 762

On the basis of participants’ explanations of their experiences, we identified their common main concern and a core category that accounted for how they worked to resolve their concern [18]. Participants described their daily experiences as requiring them to reevaluate their physical capacity and ability to work physical activity into their lives. Reconciling relationships with physical activity (the core category) involved 3 phases: 1. Gauging, 2. Engaging, and 3. Adjusting (Fig. 1). Through ongoing processes of reconciling, women discerned forms and ways of achieving physical activity they felt worked for them at various points in the postpartum transition. Women gauged the physical activities they were comfortable pursuing, and sought to achieve them through engagement strategies of pushing for, holding back, and holding still. Women adjusted their physical activity pursuit in response to physical activity experiences, most often in ways aligned with their engagement strategy: women pushing for activity pushed further or scaled back, women holding still loosened or tightened limits and women holding still gained momentum or disengaged from pursuing physical activity.

Women defined physical activity as including not only types of activities but also activity qualities, such as intensity (high, low), frequency, environment (e.g., social, independent, outdoors, indoors), and structure (e.g., flexible, spontaneous, scheduled).

Physical activity desires reflected activities that women regarded positively, specifically those activities they viewed as meeting their needs and supporting personal well-being. Women differed in associating physical activities with being central to their identity and level of well-being. For example, some women regarded physical activity as integral to their well-being: “After having a baby, you’re kind of A: desperate for some time alone and B: something to make you feel like who you were before. For me, activity is one of those things and it is a good mental break” (30). Women varied on their views of desirable physical activity engagement from broad (e.g., a mix of activities, intensities, and environments) to narrow (e.g., walking) or explicit (e.g., high intensity rock climbing) desires. Whether practiced outdoors and/or indoors, women desired independent physical activity (activity by themselves). A small group desired sports-based physical activity.

### Gauging

The participants gauged their personal capacity, relationships, and resources to determine if pursuing desirable physical activities was workable and the likelihood that pursuit would disrupt their ability to meet their own and others’ needs.

### Personal gauging

Women reported a rollercoaster of emotions and difficulty accomplishing their daily activities in limited time with limited predictability. Everything required a “lot more planning” (8) and flexibility. Women emphasized their fatigue resulting from infant night waking and described experiences of pain (e.g., hip, incisional pain), discomfort (e.g., heavy breasts), or birth/postpartum-related injuries (e.g., shoulder injury from lifting their infant). Given such problems, participants gauged how pursuing physical activities important to them would affect their vulnerability to injury, fatigue, embarrassment, or functional impairment.

Assessing personal vulnerability to negative physical outcomes (e.g., injury, fatigue) was complicated because participants described their lack of knowledge and the absence of care provider advice about returning to physical activity:

At 6 weeks, my OB said, ‘Yeah, you’re good to go. Everything looks fine.’ But my stitches looking fine does not mean that I’m actually … how does that relate to the rest of my body being able to undertake any sort of physical activity? (11). Women gauged their physical readiness for physical activity based on their previous level of fitness and/or the intensity of their physical activity desires, which led to some women gauging themselves as physically unable to perform their previous activities. “Physio told me not to do sit-ups and stuff … Which is a big part of a lot of the [fitness] classes that I was doing … I’ll just do other things” (14).

Maintaining physical functionality was important for women in gauging capacity: “I’m still not ready to engage in those activities that will bring me back more tired … I can’t injure my back, I can’t injure my arms, I have to be able to hold her” (16).

Physical activities of long duration and intensity e.g., boot camps, were forms of physical activity women were most likely to gauge as increasing their vulnerability to further injury or difficulty with daily activity.

Women indicated that structured or planned physical activities, e.g. recreational programs, were generally beyond their organizational capacities in the early postpartum period (e.g., < 6 weeks postpartum):

Even getting them out. Let’s go for an hour-long walk. That took me a while. I either needed to sleep so that I would not be crazy on four hours of sleep, or eat something … .the basics had to be done first before I could kind of take on that other piece (19). 330

Women who worried about being judged or embarrassed viewed group physical activity as emotionally unsafe. Participants who perceived reduced fitness levels gauged physical activity in social settings as increasing their vulnerability to judgment or embarrassment. “I will be hesitant to ski with people that I used to ski with because I will worry about slowing them down” (5). When participants had felt “judged by other mothers” (9) about their infant’s development and mothering practices, they limited their options by avoiding mother-infant classes and activities.

### Relational and structural workability

The environment and infant age affected participants’ perspectives about the workability of outdoor physical activities. Fewer paths and connected roadways made suburban environments less workable for walking. Infant safety limitations, (e.g., no sunscreen or jogging strollers before 6 months), older infants (who were more difficult to carry), and uneven roadways or space for strollers reduced the workability of hiking or jogging with infants.

Regardless of their income levels, participants viewed low-cost or free physical activity options (e.g., shop and stroll) as more workable. Participants described many facility childcare offerings as too restrictive (e.g., costing between $5.00–6.00 a session per child, restricting hours, and limiting infant spots), and/or mother-infant programs that excluded infants once they were crawling and older siblings. Women with limited childcare options generally found recreation centre activities unworkable. “I can’t afford to always hire a babysitter … it’s just not going to happen … A lot of rec centres, the daycares are only available from nine to noon or something, and the program I wanted was at four p.m.” (12).

Women were less likely to achieve their physical activities when they found accessing information about physical activity options or resources difficult. “They claimed to have a childminding service. I’ve seen signs, like they say that there’s childminding for certain swim times, and I’m like, where, where is this?” (17). Women with a history of fitness program engagement and peer support networks gauged more forms of physical activity as workable:

I’m lucky that I have a bunch of friends who are moms … that I have used as a resource … they’re all really active women. A couple weeks ago I said, ‘okay, so here’s my interview question for today everybody’. ‘How do you fit in exercise, what does that look like? And how do you do it’, so that I could figure out ways that I could do the same thing (30). 366

Daytime activities tended to be gauged as more workable because evening exercise could interfere with family time and infant routines: “I don’t want to go out every single night to do this [gym class]. I want to spend time with my husband. That’s fostering those relationships, too” (8).

Women gauged independent physical activity as unworkable when they were worried about the quality of their childcare options, potential effects on their infants or others, and selfishness: “I feel guilty towards my daughters for making them babysit while I’m doing something fun or something that, in my mind, I think, it’s not an essential thing” (7). Only women with adequate finances and childcare who believed exercising apart from their infants would be beneficial for everyone regarded independent physical activity as workable. “Her dad’s really good with her … I think it’s nice for her to spend just some time with her dad and I don’t want to have to spend every hour with her” (6).

### Engaging

**Fig. 2:**
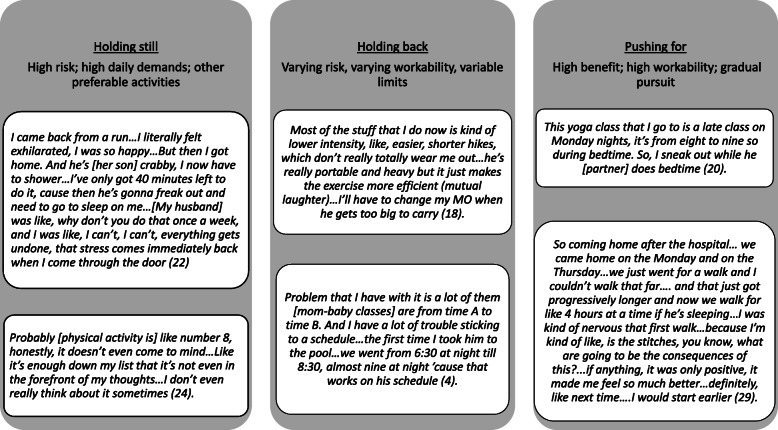
Diagram illustrating categories of engaging 764

Women engaged by using strategies of ‘holding still’, ‘holding back’ or ‘pushing for’ to pursue physical activities they gauged as workable and minimizing their own or others’ vulnerability to negative personal and relational outcomes (Fig. 2).

### Holding still

Participants who viewed pursuing most of their desired physical activities as increasing personal and relational vulnerability held still; “I just get to be mommy pretty much, no gym, nothing concerted” (21). They engaged in physical activities intrinsic to daily life (e.g., walking and caring for their baby; family walks or swims) that were important to them for supporting family relationships and occupying their time: “The kids make me get out of the house … I just took to walking … just as something to do with him, just to get him out of the house” (17). Women who viewed physical activity as less central to their well-being tended to hold still. “I think it’s probably more, it’s more stuff around the house that is like a bigger priority actually … I don’t get really antsy if I don’t get any exercise, but I get really antsy if my house is falling apart” (26). By holding still on pursuing preferred physical activities (e.g., kayaking, swimming), women saved time and energy to achieve activities they valued most (e.g., church, socializing, sewing) and were workable. “Music for me, we’ll, prioritize it and we’ll make it happen … You kind of just overcome the obstacles … with exercise, it takes a lot more mental energy to make it happen” (23).

### Holding back

Participants held back when desired physical activities exposed them to negative consequences or were regarded as unworkable; they pursued some forms of physical activity but with limitations. They believed that holding back was temporary and expected to engage fully in their desired physical activities after their infants’ care needs diminished. “It’s something that I’ll put on hold now, and get back into it when I have more time. That’s not to say that I’m not being active, just not that [type of] physical activity” (19). Participants generally pursued low-moderate intensity physical activity during the day to accommodate their infants’ schedules; they preferred flexible options i.e., drop-in sessions, rather than scheduled activities. Women varied in their boundaries for physical activity, with some avoiding group-based, indoors, scheduled and/or cost-based programs. Women were more likely to attend mother-infant physical activity programs when they viewed paying for childcare or having others care for their children as contributing to infant or relational vulnerability. “The only option … would be in the evening when my husband is not working and my older daughter is at home but then I would feel guilty cause they just came [home] and I’m doing something wrong” (1).

### Pushing for activity

Participants who gauged their physical activity desires as workable, because they supported acceptable levels of personal vulnerability, pushed for activity. They explored levels of fitness, negotiated schedules, and acted opportunistically. To push for desirable activities, these women began with lower intensity physical activities within their abilities and then progressively increased the intensity and duration of physical activities. “I feel much more fit now. I don’t have to stop and catch my breath and feel nauseous or anything. It just took a few months of like every week practice and boot camps” (6). To work out relationally and practically how to achieve desirable independent physical activities, participants explored their physical activity options (e.g., facility schedules), negotiated with others to coordinate childcare (as needed), and worked around infants’ schedules:

We’re still working out how to get the best exercise … Between 6 and 7 … I put her down on his [her husband’s] chest and I go for a run … the next challenge for us is to get her to maybe take it [a bottle] so that I can buy myself a bit of extra time (28). 438.

### Adjusting

Participants adjusted their strategies when they encountered discrepancies between their gauges of physical activity capacity and their experiences with physical activity, which were often at odds. They expressed surprise at reduced levels of fitness compared to their pre-pregnancy levels. “I was just like, oh, I’ll just run—like—I can walk a hundred miles. No. Walking around the block and I was exhausted” (11). Early negative physical activity experiences, such as embarrassment or injury, were particularly likely to prompt adjustment. A woman adjusted to walking only after experiencing injury from pursuing physical activities: “I ended up having an umbilical hernia …. I join [ed] activity way too soon, I joined back my baseball team … I was trying to go out and jog, all at 6 weeks postpartum” (13).

Participants predominantly stayed with their engagement strategy in the adjusting phase but shifted the ways they were engaging. When participants *pushing for* activity found their physical activities were working and achieving positive outcomes they pushed further. Women who felt overwhelmed or experienced persistent difficulties, e.g., childcare negotiations, scaled back their efforts to achieve physical activity. “We’ve stopped relying on my mum for childcare …. There’s always these weird strings attached … I come out of the helping with the kids worse off. I’m now full of stress from all the other stuff that came with it” (15).

Women who *held back* loosened their limits when the workability and compatibility of desirable physical activity with daily activities improved. For example, a woman who gained confidence in managing her children in a variety of situations described ‘braving’ more frequent walks and hikes: “My comfort level’s gained with it.

I’m much more able to go and do things …. I’ve been trying to challenge myself with that too … think of scenarios before, to talk myself through that ‘yes I can do this’” (19). When women holding back encountered physical or scheduling difficulties they tightened limits and reduced their physical activity even further, e.g., avoiding certain programs.

Only one woman, who was *holding still* and experienced increased vulnerability when her husband was away at work, adjusted by disengaging from physical activity pursuit. Women who previously held still gained momentum and more intentionally pursued their physical activity desires when they viewed physical activity as essential for their well- being. A woman expressed her thoughts about increasing activity after trying yoga: “I think I should change my mind and put one hour for doing exercise …. I mean, mind and my stress, it is good, and my body, both of them” (3).

### Outcomes of reconciling

By reconciling their relationships with physical activity, most women were comfortable that their postnatal physical activity choices fit their own and others’ needs and resources. Nonetheless, their comfort included both positive feelings and dissatisfaction about their experiences. A woman described the tension between her positive feelings and the limitations in her physical activity options.

I find [when] I can get some physical activity in with her, she sleeps better … I feel better and I sleep better, and I have more energy and I don’t feel guilty. I wanna do physical activity …. We also try to find activities that are also beneficial to the kids, so it’s kind of a compromise, like, would I go and splash around by myself [at the pool], probably not (25). Women holding back and holding still remained hopeful that they could better align physical activity patterns with their desires but believed that having a child (or children) affected opportunities for physical activity. For example, women holding back described the need to “let go” (19), live their “new normal” (16), and be “satisfied with less” (5); “It’s just finding a different activity that fits … I think it’s just acknowledging that it’s going to be different things than what it was before” (11).

Women maintained the same strategy of engaging unless they were in the group pushing for activity. Two women who were pushing for activity adjusted to holding back when they experienced emotional satisfaction with motherhood that was beyond the benefits physical activity could provide for their well-being “*Interviewer*: And what makes it okay that you can’t get the physical activity you want; *Participant*: The fact that I’m so in love with my daughter” (16). Two other participants who were pushing for activity expressed anger and frustration from feeling they had to adjust to holding still and sacrifice their emotional health and feelings of accomplishment because of injury and difficulties with childcare: “I miss going to the gym, I enjoy doing that. I enjoy going for runs …. Landlocked, that’s it …. I feel more unhappy now because it sucks. I don’t look as good or feel as good as I used to” (13).

## Discussion

The current study provides an original integration of how women perceive diverse personal, relational, and environmental factors affecting their physical activity choices following birth. Regardless of their previous experiences and number of children, the postpartum transition required study participants to revisit their capacities for desirable physical activities and their workability. Similarly, English women have described social pressure and physical and psychological changes during pregnancy and early postpartum affecting their thoughts about how to retain ownership of their bodies in making physical activity decisions, with limited trustworthy information [17]. In the current study, the processes involved in reconciling relationships with physical activity explained women’s postpartum reconfigurations of physical activity on personal, relational, and pragmatic levels. Lloyd et al. [21] presented the postpartum period as a critical juncture where women navigate the subjectivities of their embodied relations to self and the meaning of physical activity.

Reconciling relationships with physical activity highlights how physical strengths and limitations intersect with postpartum physical activity decision- making. Women’s perceptions of their capacity for physical activity were influenced by pain, injury, fatigue, energy, physicality of infant care, and perceived fitness. Participants often described ongoing pain, fatigue, or injury that affected their physical activity engagement. These findings suggest that women may encounter major physical impediments to engaging in physical activity following birth. Physical considerations are consistent with other postpartum literature; women reported difficulty and disappointment regaining pre-pregnancy fitness levels, with up to 70% of women in the year following birth reporting at least one physical symptom (e.g., back, hip, and pelvic pain), and 45% linking symptom(s) with moderate or severe functional impairment [22, 23].

Most study participants expressed difficulty gauging appropriate physical activity engagement due to uncertainty about their personal capacity. Consequentially, women described experiences of over or underestimating their personal capacity for physical activity. When participants reported negative emotional and physical consequences (e.g., experiencing injury or feeling overwhelmed with the planning to achieve physical activity), their gauges of personal capacity did not match their experiences; they usually downgraded their physical activity pursuits in response. Other participants recognized that they could have returned to physical activity earlier following birth. Women have described being cautious with physical activity engagement during pregnancy due to uncertainty about the effects of physical activity on their infant [17]. Our study findings suggest uncertainty about personal capacity postpartum can unduly contribute to cautious physical activity engagement for women.

Consistent with built environment and sociological physical activity literature [20] study participants highlighted structural features (e.g., physical environment, recreation program availability) and resources (e.g., childcare, finances) in their decision-making about physical activity. Women in previous studies [12, 13] have also reported a number of environmental barriers to physical activity following birth. The current study extends the literature because participants described unique postpartum environmental challenges, such as the safety of navigating the outdoor terrain with strollers, lack of recreation centre family-inclusive programming, and need for flexibility in program delivery and childcare options.

Participants’ descriptions of difficulty accessing information about postpartum physical activity programming and recreation centres’ childcare aligns with previous work indicating women have perceived limited access to relevant physical activity resources and information for the postpartum period [12, 13]. Our findings point to the importance of available information for postpartum physical activity decision-making, because study participants were less likely to pursue physical activity when they lacked confidence to navigate recreational programming information or access to social networks of people who could support their strategizing.

Women in the current study had varied interpretations about physical activity options, suggesting the postpartum physical activity barriers identified in the literature (e.g., lack of time, childcare) are not equally relevant for all women. Participants who regarded physical activity as central to their well-being and identities pushed for physical activity. Our findings fit with leisure science that positions constraints as relative to peoples’ interpretations; with higher motivation, people will engage more with personal, relational, and structural constraints and strategize how to access their desired leisure [24]. In the current study, women with childcare options described negotiating with others for childcare to support high priority activities they viewed as workable and low-risk. Only participants who emphasized the importance of independent physical activity as both integral to their well-being and low risk to others were comfortable pursuing it.

Participants varied in desires for physical activities intensities and settings. Variations in women’s physical activity desires and interpretations of workable and achievable physical activity suggest that postpartum physical activity interventions designed to address generalized barriers (e.g., free mother-infant classes) and facilitators (e.g., planned outdoor activities) may have limited capacity to contribute to women’s increased physical activity participation. Pursuing mother-infant fitness programming has been recommended to facilitate women’s postpartum physical activity [12, 13] however, in the current study, some participants gauged themselves as emotionally vulnerable in such environments. Tailored and extended physical activity support that encourages women to identify and strategize to achieve desirable physical activities has more successfully supported sustained increased in physical activity than generalized support [25].

Tailoring support to women’s engagement strategies or orientation towards attaining postpartum physical activity could be an effective approach for women’s achievement of their physical activity desires. In the current study, most women adjusted within their chosen engaging strategy. These findings align with longitudinal research indicating relative stability in women’s nutrition and weight orientations (attitudes and patterns) from pregnancy across the postpartum period [26]. Although women in our study generally maintained the same approach to pursuing physical activity, they expressed tension about their physical activity choices. Studies exploring relationships between patterns of reconciling and women’s well-being could describe pathways linking physical activity decision-making, mental health, and well-being across the postpartum transition.

### Limitations

Participants were English speaking, and most, highly educated with incomes >$80,000 (similar to local median incomes between $76,000-93,000), which limits applicability of study findings [27]. The study data were collected in 2014–2015, which represents a particular context that may have changed in the intervening years.

Environmental safety has been linked with physical activity choices [20], but these study participants did not report safety considerations (e.g., crime). Incontinence may affect physical activity choices and is common for women following birth [28], but the study participants did not disclose this.

## Conclusion

Reconciling relationships with physical activity is an original theoretical representation that explains women’s physical activity decision-making processes. Study participants were continually reconciling diverse considerations to discern the extent to which they were comfortable pursuing desirable physical activity. They tended to downgrade their aspirations when encountering challenges, particularly after experiencing injury or extensive difficulties finding childcare. Without support to return to physical activity during the postpartum period, it is difficult for women to sustain sufficient motivation and perceptions of their capacity to pursue physical activities that they identify as supporting their own and families’ health.

## Supplementary Information

**Additional file 1:.** Interview guide. Guide used in initial interviews

## Data Availability

The data that support the findings of this study are available on request from the corresponding author. The data are not publicly available due to privacy or ethical restrictions.
