# Supplementary material for: Reconciling relationships with physical activity: a qualitative study of women’s postnatal physical activity decision-making
Source: BMC Pregnancy Childbirth. 2021 Jan 25;21:81. doi: 10.1186/s12884-020-03537-z (PMC7831196; doi:10.1186/s12884-020-03537-z)
Supplement: Supplementary file 1 — Additional file 1:. Interview guide. Guide used in initial interviews [file 12884_2020_3537_MOESM1_ESM.pdf]

Initial interview guide

Research question: What are women's decision-making processes about physical activity in the postnatal period?

Introduction[1]

- Review and discuss their diary; discuss participant's perceptions of physical activity.
- What does physical activity mean to you? (i.e. what do you consider as physical activity)

General

- What are your experiences so far with physical activity since you have had your child? (If mother has other children) How do these experiences compare with your last child?
- What are the thoughts you have had about physical activity since you have had a child?
- How have your thoughts about physical activity changed (if at all) since having your baby?
- What are some of the things that need to happen for you to be able to be physically active?
- How do you feel when you do physical activity? Before (planning), during, after?
- How do you think about physical activity in your day-to-day life?
- What motivates your engagement in physical activity?
- Do you have goals around physical activity? What helps or hinder you to achieve your goals around physical activity?

Wrap-up

- Do you have anything to ask me?
- Is there anything else you can think of that you might not have thought about before to add?

[1] Interview structured via framework laid out in:

Tod, A. (2006). Interviewing. In K. Gerrish & A. Lacey (eds). *The Research Process in Nursing* (5th Edition). Blackwell Publishing.
